# Bone Turnover Markers After Six Nights of Insufficient Sleep and Subsequent Recovery Sleep in Healthy Men

**DOI:** 10.1007/s00223-022-00950-8

**Published:** 2022-02-08

**Authors:** Christine M. Swanson, Prajakta Shanbhag, Emma J. Tussey, Corey A. Rynders, Kenneth P. Wright, Wendy M. Kohrt

**Affiliations:** 1 Division of Endocrinology, Metabolism and Diabetes, Department of Medicine, University of Colorado Anschutz Medical Campus, Aurora, CO, USA; 2 Division of Geriatric Medicine, University of Colorado Anschutz Medical Campus, and Eastern Colorado VA Geriatric, Research, Education, and Clinical Center (GRECC); Aurora, CO, USA; 3 Sleep and Chronobiology Laboratory, Department of Integrative Physiology, University of Colorado Boulder, Boulder, CO, USA

**Keywords:** Sleep restriction, bone turnover markers, insufficient sleep, recovery sleep

## Abstract

**Purpose:**

The goal of this study was to determine the bone turnover marker (BTM) response to insufficient and subsequent recovery sleep, independent of changes in posture, body weight, and physical activity.

**Methods:**

Healthy men (N = 12) who habitually slept 7-9 h/night were admitted to an inpatient sleep laboratory for a baseline 8 h/night sleep opportunity followed by six nights of insufficient sleep (5 h/night). Diet, physical activity and posture were controlled. Serum markers of bone formation (osteocalcin, PINP) and resorption (β-CTX) were obtained over 24-h at baseline and on the last night of sleep restriction, and on fasted samples obtained daily while inpatient and five times after discharge over 3 weeks. Maximum likelihood estimates in a repeated measures model were used to assess the effect of insufficient and subsequent recovery sleep on BTM levels.

**Results:**

There was no statistically or clinically significant change in PINP (*p* = 0.53), osteocalcin (*p* = 0.66) or β-CTX (*p* = 0.10) in response to six nights of insufficient sleep. There were no significant changes in BTMs from the inpatient stay through three weeks of recovery sleep (all *p* ≥ 0.63). On average, body weight was stable during the inpatient stay (Δweight = −0.55 ± 0.91 kg, *p* = 0.06).

**Conclusion:**

No significant changes in serum BTMs were observed after six nights of insufficient or subsequent recovery sleep in young healthy men. Changes in weight and physical activity may be required to observe significant BTM change in response to sleep and circadian disruptions.

## 1.0. Introduction

Insufficient sleep duration is a risk factor for many cardiometabolic diseases including diabetes mellitus type 2, obesity, coronary heart disease and all-cause mortality [1–11]. Emerging literature, primarily from pre-clinical models, suggests insufficient sleep duration may also influence bone metabolism [12,13]. Two studies of chronic sleep restriction in rats demonstrated declines in markers of bone formation with either no change or an increase in markers of bone resorption [13,12]. As might be expected from this detrimental uncoupling of bone turnover markers where resorption exceeds formation, these studies also demonstrated lower bone mineral density (BMD) and poorer bone microarchitecture in sleep-restricted rats compared to controls [12,13]. In humans, cross-sectional studies have identified associations between short sleep duration and low BMD [14–17]. The Women’s Health Initiative (WHI) identified increased fracture risk with sleep duration of ≤5 hours/night [18]. Although associations have also been identified for long sleep duration with both low BMD and fracture risk [18,19,17], short sleep duration is more commonly experienced. In fact, the CDC highlighted insufficient sleep as a public health epidemic in 2014 [20], with over one-third of U.S. adults reporting less than 7 hours (h) per night [21]. Prior human intervention studies identified a decline in serum markers of bone formation in response to severe sleep restriction (2 h/night) [22] and, in a separate study, three weeks of cumulative sleep restriction (~5.5 h per 24 h) with concurrent circadian disruption [23,24]. However, the effects of modest, commonly experienced insufficient sleep duration (e.g., 5 h/night), mechanisms by which insufficient sleep affects bone metabolism, and the ability to recover from these exposures with sleep extension had not been studied.

The goal of the present study was to determine the bone turnover marker (BTM) response to sleep restriction (5 h/night for six nights) and subsequent recovery sleep, independent of changes in body weight, posture, or physical activity. Based on our prior work [23,25] and animal data [12,13], we hypothesized that six nights of insufficient sleep duration would cause declines in serum markers of bone formation (osteocalcin; N-terminal propeptide of type I procollagen, PINP) but not a marker of bone resorption (C-telopeptide of type I collagen, β-CTX) and that bone formation marker levels would return to baseline within three weeks of resuming a normal sleep/wake schedule.

## 2.0. Methods

### 2.1 Study Design and Participant Selection

Participants were recruited from the Denver, Colorado area between January 2019 and January 2020 using flyers, electronic advertising, and social media marketing. The protocol was performed in twelve healthy men who habitual slept 7-9 h/night and consisted of three consecutive phases: outpatient habitual controlled sleep duration during a run-in week, inpatient baseline night followed by sleep restriction, and observational recovery phase. The protocol was performed in men because when the study was designed, data demonstrating changes in BTMs in response to sleep interventions were available only in men [23,26] and male animals [12]. All men were healthy as determined by questionnaires, labs (including a urine drug screen), BMD screening by dual energy x-ray absorptiometry (DXA), history and physical exam prior to study entry. All men were screened using the Berlin questionnaire and a WatchPat [27] home sleep apnea testing device to exclude for moderate to severe sleep apnea. Participants were free of self-reported clinically significant sleep disorders or medical conditions and took no prescription medications.

To be eligible for the study, participants could not routinely go to bed after midnight, have a history of night shift work in the past year, have lived outside the Denver area (i.e., at altitude) in the past 3 months, or have traveled >1 time zone away in the past 4 weeks.

**Figure 1:**
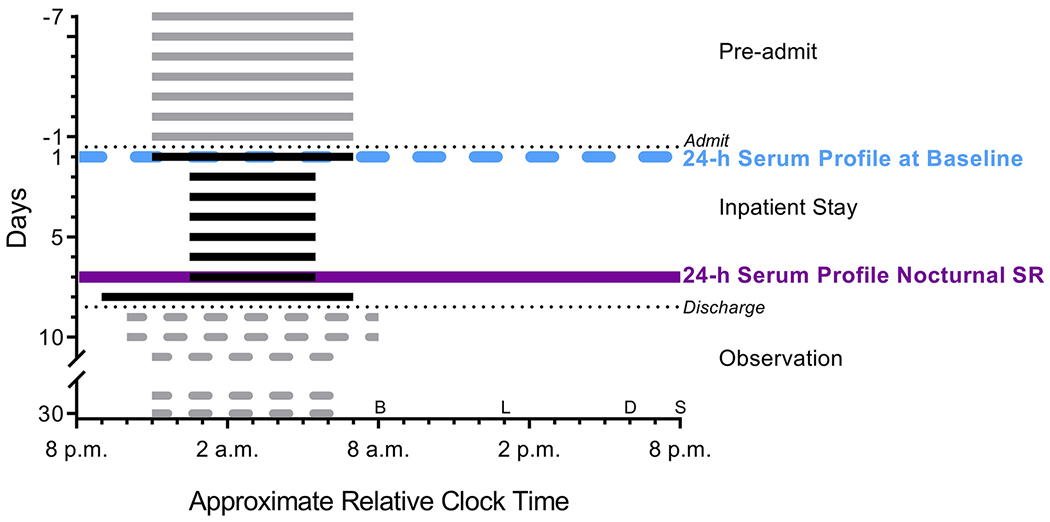
Study Protocol X-axis represents approximate relative clock time based on average bedtime and waketime of participants self-selected 8 h sleep opportunity. Horizontal grey bars represent outpatient 8 h sleep opportunities prior to admission. Horizontal black bars represent inpatient sleep opportunities. Horizontal dotted grey bars represent theorized sleep schedules during 3-week outpatient observational segment of the protocol with catch-up sleep in the days after discharge and then resumption of habitual sleep/wake schedule. Dotted black lines represent when participants were admitted to and discharged from the inpatient stay. Dotted light blue horizontal line indicates baseline 24 h serum collection. Solid purple horizontal line represents 24 h serum collection during nocturnal sleep restriction (SR). Letters along the x-axis represent inpatient meal times for breakfast (B), lunch (L), dinner (D) and snack (S).

During the outpatient habitual sleep duration run-in phase, all participants maintained a consistent 8-h sleep opportunity at their habitual bed/wake time, verified by wrist actigraphy, sleep diary and time-stamped texts (Figure 1). Immediately following the outpatient week, participants were admitted to the University of Colorado Clinical and Translational Research Center (CTRC) inpatient sleep suite for an 8-night inpatient stay. The first night was a baseline night with a habitual 8-h sleep opportunity, during which blood was obtained hourly for 24 hours. On nights 2-7, participants were sleep restricted to a 5-h sleep opportunity per night. Sleep was shortened by delaying bedtime and advancing wake time by 1.5 h each. On night 7, the hourly blood draws were repeated for 24 h, at the same time points as on night 1. All 24-h blood profiles were aligned across and within participants to the midpoint of sleep. Fasted blood was also collected on days 2-6 and on day 9 at habitual wake time. On nights 2-7 when sleep restriction was imposed, participants remained semi-recumbent (head of bed raised 35°) for the 1.5 h before bedtime and 1.5 h after wake time to control for postural changes between conditions that may contribute to BTM changes, while helping to maintain wakefulness. For safety, participants were provided with a ≥10 h sleep opportunity after the last 24-h blood draw was completed on night 8 prior to discharge on day 9. Participants had 24/7 supervision during both 24-h blood draws and constant supervision while awake throughout the inpatient phase to ensure adherence to the protocol. There was one protocol deviation in which one participant slept ~1 h longer than prescribed on night 5 of sleep restriction.

After discharge from the inpatient unit, participants could opt in for an observational recovery phase to investigate the potential time course of reversibility of sleep restriction-induced changes in BTMs. During this 3-week observation phase, participants wore the physical activity tracking devices and maintained a sleep log but were not given instructions on sleep duration or timing. Morning fasted blood was to be obtained on days 1, 2, 6, 13 and 20 after discharge, but approximately 33% of these draws occurred on different days due to scheduling issues. One participant declined to participate in this portion of the protocol. Three of the men missed one draw each due to scheduling issues and the COVID-19 pandemic.

All participants provided informed consent. The protocol was approved by the Colorado Multiple Institutional Review Board (COMIRB) and registered at ClinicalTrials.gov (NCT03733483).

### 2.2 Participant Characteristics

Race/ethnicity was self-reported using the standard NUT categories. Body mass index (BMI) was calculated using height and fasted weight obtained at the screening visit using a stadiometer and digital scale. Serum calcium and creatinine were measured at the University of Colorado Hospital clinical and reference laboratory using indirect ion selective electrode and colorimetry, respectively, at the screening visit to evaluate for underlying abnormalities that could affect study results (e.g., chronic kidney disease). To qualify for the study, participants’ calcium and creatinine at the screening visit had to be within the lab’s normal range (8.6-10.3 mg/dL and 0.70-1.30 mg/dL, respectively) with an estimated glomerular filtration rate (eGFR) ≥ 60 mL/min/1.73 m^2^ using the IDMS-traceable CKD-EPI equation for ages 18-97 years old used by the University of Colorado clinical laboratory. To be enrolled in the study, participants had to have a negative urine drug screen at both the screening visit and inpatient admission.

### 2.3 Wrist Actigraphy and Outpatient Sleep Data Collection

Bedtime, wake time and sleep duration were tracked in multiple ways during outpatient phases. All participants wore a wrist actigraphy device (ActiWatch-2 [28]) on their non-dominant wrist before, during, and after admission to track sleep/wake and physical activity levels and patterns. Participants were instructed to push the ActiWatch2 event marker at bed and wake times. Participants also completed a sleep diary during outpatient phases. During the outpatient run-in phase, bed and wake times were also verified with time-stamped, secure texts. For time-stamped texts, participants texted “bedtime” or “wake time” to a secure Google voice phone number when they went to sleep and woke up, respectively. These texts were stamped with the time they were sent and were used to track each participant’s sleep timing and duration and to ensure adherence to the outpatient protocol. Average bed and wake times reported via text from the run-in week were used to determine habitual bed and wake times for the inpatient week.

### 2.4 Polysomnography

During the two 24-h blood collection intervals, sleep was verified by the Siesta 802 polysomnography device (Compumedics USA Inc). Polysomnography electroencephalogram (EEG), bilateral submental electromyographic (EMG) and bilateral electrooculographic (EOG) electrodes were applied by study staff using tensive adhesive gel. The following EEG channels were obtained: F3/A2, C3/A2, C4/A1, and O1/A2. CZ and FZ were used as reference and ground electrodes, respectively. Polysomnography sleep stages were scored by one trained scorer using AASM guidelines [29].

### 2.5 Bone Turnover Markers (BTMs)

Blood was processed immediately after collection and stored at −80°C until thawed for assays. All samples from an individual were run in the same assay to minimize variability. C-telopeptide of type I collagen (β-CTX) was used as a marker of bone resorption. Osteocalcin, and intact N-terminal propeptide of type I procollagen (PINP) were used as markers of bone formation. Serum samples were assayed in singleton at the University of Colorado CTRC Core laboratory, which provided inter- and intra-assay coefficients of variation (CV). β-CTX, PINP and osteocalcin were measured by chemiluminescence using the automated IDS iSYS instrument (Immunodiagnostics Systems, United Kingdom). For β-CTX, interassay CV was 8.6% at 196 ng/L and 6.4% at 406 ng/L and 5.8% at 2,080 ng/L. β-CTX intra-assay CV was 7.7% at 201 ng/L, 4.6% at 446 ng/L, and 3.4% at 2,050 ng/L. PINP interassay CV was 5.6% at 20.36 *μ*g/L, 3.5% at 51.23 *μ*g/L and 1.7% at 175.21 *μ*g/L. PINP intraassay CV was 4.5% at 2.77 *μ*g/L, 1.9% at 37.24 *μ*g/L and 2.0% at 175.84 *μ*g/L. Osteocalcin inter-assay CV was 1.9% at 7.95 ng/mL, 3.9% at 61.73 ng/mL and 5.5% at 138.20 ng/mL. Osteocalcin intra-assay CV was 4.0% at 2.72 ng/mL, 1.7% at 23.70 ng/mL and 2.4% at 137.65 ng/mL.

### 2.6 Heparin

Low dose heparin (4,000 units/L normal saline) was used to facilitate overnight blood draws through a long intravenous (IV) line that passed through a port in the wall of the inpatient room while the participant was sleeping so as not to disturb sleep. A blood sparing sampling method with Vamp syringe tubing was used for blood draws performed with the long IV tubing (volume 5 cc) with multiple sampling ports. With this procedure, 10 cc of “waste” were drawn through the long IV tubing into a syringe, the blood sample was subsequently drawn from another port located closer to the participant’s IV site, and after the sample was obtained the 10 cc of “waste” were returned to the participant. The long IV tubing was flushed for over one minute (~20 cc of saline) to completely clear the line of any residual heparin before the sample was drawn and again after the sample was obtained, before the heparin was turned back on. Heparin and long IV tubing were only used during sleep opportunities. Therefore, heparin exposure was three hours longer during the baseline 24-h collection compared to the sleep restricted 24-h collection.

### 2.7 Sleep Questionnaires

All participants completed the Pittsburgh Sleep Quality Index (PQSI) [30] during screening to evaluate for subjective sleep complaints. Participants were excluded based on a global PSQI score >5 indicating poor sleep quality. During the study, subjective sleepiness was assessed with the Karolinska Sleepiness Scale (KSS) three times a day (30 minutes after habitual wake time, 5.5 hours after wake and 30 minutes before habitual bedtime). The Epworth Sleepiness Scale [31,32] was performed at screening (during which individuals with a score >10 were excluded), the first day of the outpatient phase and on days 1, 2, and 8 of the inpatient phase.

### 2.8 Physical Activity

Physical activity levels and patterns for a given individual were matched during their outpatient and inpatient weeks using wrist actigraphy (ActiWatch2) and a Fitbit Charge 3 device to monitor for differences in physical activity that could confound the results. In addition, physical activity was standardized between individuals by having each participant perform three, 20-minute walking bouts daily during the outpatient run-in and inpatient weeks. To ensure protocol adherence during the outpatient run-in week, participants texted the same secure, time-stamped phone number that was used to track sleep times when they completed their daily walking bouts. Physical activity (in arbitrary activity counts) was tracked with the ActiWatch-2 [28]. Actigraphy data were cleaned and analyzed according to the MESA scoring manual [33]. Participants also wore a Fitbit Charge 3 device to track step counts to facilitate physical activity matching between outpatient and inpatient weeks by allowing for remote monitoring and check-ins throughout the day. Fitbit data from the outpatient run-in phase were used to match participant step counts (±10%) for average weekday and weekend days. In addition, workouts participants completed during the outpatient run-in week were recorded and matched during the inpatient week using the research gym attached to the inpatient unit.

### 2.9 Diet

Meals were provided by the CTRC Nutrition Core for the last three days of the outpatient week leading up to admission and throughout the inpatient stay. Caloric needs were determined using the Mifflin-St. Jeor Equation for men based on a participant’s age, height, weight and an activity factor of 1.4-1.7 (varied depending on participant’s self-reported activity and Fitbit-recorded activity during the outpatient run-in week) [34]. Participants received their calories in three meals (breakfast, lunch, dinner) and an after-dinner snack (Figure 1). Participants received 7% more calories on sleep-restricted days to compensate for increased energy expenditure [35,36], which were distributed across all meals. Three men required an additional food during sleep restriction due to hunger. Fasted morning weights were obtained daily at habitual wake time, after the fasted morning blood draw. Macronutrient content was 55% carbohydrate, 30% fat and 15% protein. Diet was controlled for calcium (1,000 mg ± 100 mg per day) and phosphorous content (1,600 mg ± 100 mg per day). Meal times were standardized to occur at 12 h (breakfast), 17 h (lunch), 22 h (dinner) and 24 h (snack) into the sampling profiles during the inpatient phase. Caffeine was not permitted during the outpatient habitual sleep duration phase or inpatient sleep restriction phase. Participants had to report habitual alcohol use during screening and only those with ≤14 alcohol drinks per week and ≤5 drinks in one sitting were eligible to participate. Alcohol was not explicitly prohibited during the outpatient habitual sleep duration phase. However, participants were instructed to only consume food and beverages (other than water) provided by the CTRC Nutrition Service three days prior to and throughout inpatient admission. No alcohol was provided with the CTRC meals.

### 2.10 Light Exposure

No light control was included in this protocol. Lights in the CTRC inpatient unit are 28 watts in the 3500-4100 k spectrum. From the angle of gaze on the inpatient bed, the typical lux levels in the sleep suite on cloudy and sunny days were 198.7 lux and 1,200 lux, respectively. A blackout shade was used during sleep periods and lights were controlled from outside the room. During lights-out sleep periods, typical lux was 0.6.

### 2.11 Daylight Saving Time (DST)

Only one participant was studied around a DST transition. In that case, DST ended on the third day of their outpatient habitual sleep duration run-in week. To keep light exposure and circadian alignment consistent throughout the duration of the study, bed and wake times after DST ended were moved one hour earlier than habitual (e.g., 11pm habitual bedtime pre-DST end and 10pm bedtime post-DST end).

### 2.12 Statistical Analysis

Using a paired t-test and prior data [23], we had >80% power to detect a change in PINP of ≥10% (assuming 11% variability in the percent change, a correlation of 0.5 between measures on the same participant, and a 2-sided alpha = 0.05). Similar to prior analyses [23,24], maximum likelihood estimates in a repeated measures model was used to assess the effect of insufficient and subsequent recovery sleep on BTM levels, accounting for potential within-participant correlation. This was done by evaluating the test statistic F for equality of means using the ratio of the variation due to insufficient and subsequent recovery sleep to the residual variation, after between-subject variation has been removed. Change in BTMs in response to insufficient sleep was assessed with the mixed model described above using BTM values measured every two hours on two, 24-h profiles obtained at baseline and on the 6^th^ night of sleep restriction. Change in BTMs in response to subsequent recovery sleep was assessed with the mixed model described above using eight BTM values starting with one value from each 24-h serum collection through the end of the observational recovery phase. Circadian rhythmicity was included in the repeated-measures model for all three BTMs as diurnal variation has been documented in prior studies [37–39]. Data from the repeated measures model are presented as estimate ± standard error of the estimate (SEE) unless otherwise stated. Descriptive variables are presented as mean ± standard deviation (SD) or number (percent), as appropriate. Paired *t*-tests were used to determine if changes in weight, physical activity and sleep questionnaire scores were statistically significant. To assess the effect of heparin on BTM assessment, a paired *t*-test was performed on data for each BTM from the third time point of each 24-h collection profile when heparin was on at baseline but not during the sleep restricted collection. A *p*-value <0.05 was considered statistically significant. All analyses were conducted using SAS software version 9.4. All figures were generated using GraphPad Prism 9.0 (GraphPad Software).

## 3.0 Results

Men were 21-40 years old at the time of inpatient admission (average 28.3 ± 5.7 years; Table 1). Race/ethnicity were determined by self-report as follows: Caucasian/Not Hispanic (N = 6), Asian/Not Hispanic (N = 4), Black/Not Hispanic (N = 2). On average, polysomnography-determined sleep duration was 404 ± 42 minutes and 283 ± 11 minutes on the baseline and sleep restricted 24-h profile nights, respectively. Consistent with imposed sleep restriction, sleepiness increased over the course of the inpatient stay (ΔESS = 4.67 ± 6.23, *p* = 0.02). On average, participants had 7.26 ± 0.7 h time in bed per 24 h during the three-week observational recovery phase of the protocol with an average time of sleep onset of 12:13 am and an average wake time of 7:41 am.

**Figure 2:**
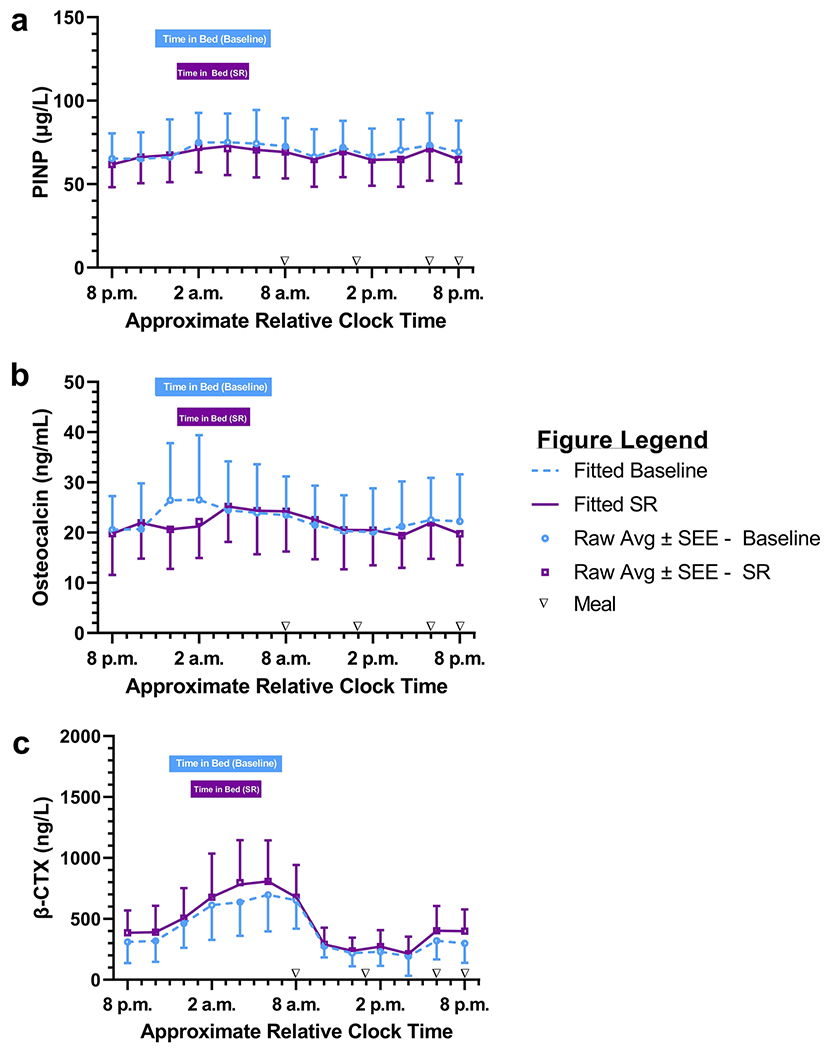
Serum BTM 24 h profiles at baseline and during sleep restriction (SR) Data from 24 h serum BTM profiles obtained at baseline (blue) and on the last night of sleep restriction (SR; purple) are displayed for (a) PINP (*μ*g/L), (b) osteocalcin (ng/mL), and (c) β-CTX (ng/L). X-axis represents approximate relative clock time based on average bedtime and waketime of participants self-selected 8 h sleep opportunity. Upside-down triangles along the x-axis represent meal times including breakfast (~8 a.m.), lunch (~1 p.m.), dinner (~6 p.m.) and snack (~8 p.m.). Each graph displays raw average values for each bone biomarker obtained every two hours as open light blue circles (baseline) or open purple squares (SR) with one-way standard error of the estimate (SEE) bars. Fitted 24 h curves are depicted as a dotted light blue line for baseline and a solid purple line for SR. Time in bed sleep opportunities at baseline and during SR are represented by horizontal light blue and purple bars, respectively, at the top of each graph.

There were no statistical or clinically significant changes in PINP, osteocalcin or β-CTX in response to six nights of insufficient sleep duration (Table 2; Figure 2). Results were unchanged when data were re-analyzed after excluding the participant who experienced the protocol deviation (*data not shown*).

Although there was no change in BTMs in response to sleep restriction, the trajectory of BTM change with resumption of normal, unprescribed sleep schedule was examined for three weeks after discharge to determine if the inpatient environment influenced BTM levels during both the baseline and post-intervention assessments. There were no significant changes in PINP, osteocalcin or β-CTX from the inpatient stay through three weeks of recovery sleep (all *p* ≥ 0.63; Table 3). Results were unchanged when the analysis was repeated after excluding the first 24-h baseline profile data point (*data not shown*).

On average, daily physical activity levels assessed by Fitbit and wrist actigraphy were slightly lower during the inpatient week compared to the outpatient run-in week (575 ± 648 fewer steps *p* = 0.01 and 11.4% ± 10.0% lower activity *p* < 0.01, respectively; Table 4). However, there were no statistically significant differences in activity level between the two 24-h collection days by wrist actigraphy or Fitbit (Table 4). On average, weight was stable from admission to the end of sleep restriction (Δweight = −0.55 ± 0.91 kg, *p* = 0.06). There were no clinically or statistically significant differences between the two means at minute 240 (~midnight in Figure 2) when heparin was on during the baseline collection but off during the subsequent sleep restricted collection in β-CTX (41.9 ± 146.8 ng/L, *p* = 0.343), PINP (1.3 ± 24.3 *μ*g/L, *p* = 0.858) or osteocalcin (5.8 ± 9.2 ng/mL, *p* = 0.053) levels.

## 4.0 Discussion

This was the first human study designed to evaluate BTM change after exposure to six nights of insufficient 5 h/night sleep duration, the mechanistic role of energy intake, physical activity and posture, and the ability of BTMs to recovery with resumption of normal sleep/wake schedules. No statistically or clinically significant [40] changes in BTMs were identified in response to insufficient sleep when energy balance, physical activity and posture were controlled, or during subsequent resumption of habitual sleep/wake schedules over three weeks.

The lack of BTM changes in response to insufficient sleep duration observed in this protocol could inform underlying mechanisms by which sleep disruption can alter bone metabolism, including the role of energy intake/weight change, physical activity, and posture during insufficient sleep. In the current study, caloric intake was increased 7% during sleep restriction to account for increased energy expenditure observed in previous sleep restriction studies [35,36]. As a result, participant weight was stable from the beginning to the end of the protocol. This differs from prior studies that identified significant differences in BTMs in response to sleep restriction and circadian disruption in humans [23,24,26,22] and animals [12] in which food intake was not increased and/or weight loss occurred with the sleep/circadian intervention. In the sleep restriction study by Xu et al, animals had access to food *ad libitum*, but sleep restricted rats gained less weight than controls [13]. This theory is consistent with another recent study that did not observe BTM change in response to nine nights of insufficient sleep with or without recovery sleep in which food intake was *ad libitum* [41]. These data are consistent with the theory that weight loss (or less weight gain) during sleep restriction contributes mechanistically to BTM changes in response to sleep restriction. Furthermore, the current study had no significant changes in physical activity level or posture between the two 24-h collection days, by design. Prior studies observed an increase in physical activity from the beginning to the end of the inpatient protocol [23,24], which may have contributed to the weight loss and BTM changes. It is possible that inherent differences in the inpatient environment in which these studies were conducted, compared to the habitual home environment, contribute to BTM changes independent of the sleep or circadian disruption imposed. However, the lack of BTM change observed from the end of the inpatient stay to the end of the 3-week observation phase in the current protocol argues against any effect of the inpatient environment itself on BTM levels in this study.

The sleep restriction imposed in the current study may not have been sufficient to provoke significant changes in bone metabolism. The magnitude of sleep restriction (5 h/night) was similar to prior human studies in which significant BTM changes were observed within the first 10 days of exposure to sleep restriction (~5.5 h per 24 h day) with concurrent circadian disruption [23,25,24]. Furthermore, a study by Staab et al demonstrated significant changes in BTMs after just one night of 2 h/night sleep restriction [22]. These data suggest that the magnitude and duration of sleep restriction in the current protocol should have been sufficient to provoke significant BTM changes. However, the previous human studies imposed more significant circadian disruption (by using a forced desynchrony protocol and significantly shorter sleep) than the current study, potentially explaining the different results. In fact, in a recent study, statistically non-significant BTM changes in response to insufficient sleep were correlated with morning circadian misalignment [41]. The 24-h profiles of osteocalcin and β-CTX appeared to briefly diverge during the prescribed sleep opportunities (Figure 2) and occurred in the same directions as observed in prior studies (i.e., osteocalcin decreased and β-CTX increased in the sleep restricted profile). The clinical significance of these brief divergences between the two 24-h profiles and their possible relationship to longer duration of heparin exposure during baseline compared to the sleep restricted day requires further research. Prior literature [42] and data from this study suggest that the very low dose heparin used only during two sleep opportunities in this study did not significantly affect bone turnover marker assays or levels.

This study to investigate changes in bone metabolism in response to insufficient sleep had several strengths including a rigorous study design, controlled inpatient environment, matching of physical activity, and dietary control resulting in stable body weight. However, there were some limitations. First, a protocol deviation resulted in slightly longer sleep duration on one night in one participant. However, results were unchanged when data were re-analyzed without data from that participant. Second, only a relatively small cohort (N = 12) of young, healthy, physically active men was studied. However, prior data [23,24] suggested the greatest magnitude of BTM change in response to sleep and circadian disruption occurred in younger individuals and *a priori* power calculations suggested the study was adequately powered to observe clinically significant changes in PINP in this racially diverse cohort. It remains possible that the study was underpowered or that the diversity of the cohort limited the ability to detect significant differences in certain subgroups. Although the skeletal response to sleep restriction has not been shown to differ by race/ethnicity [25,41,18], this should be explored in future studies. Third, the similar physical activity levels and bedrest imposed during shortened sleep may not be commonly experienced when sleep restriction occurs in real life. However, controlling these factors may provide mechanistic insight into how sleep restriction alters bone metabolism. Fourth, the controlled diet in this study may not accurately replicate real-life changes in food intake in response to sleep restriction, as previous studies have demonstrated increased caloric intake with preference for carbohydrate foods during sleep restriction [35,36]. Although calorie intake was increased during sleep restriction in the current study, participants did not have *ad libitum* access to food. However, as discussed above, this may indicate that changes in food intake and subsequent weight change play a mechanistic role in the detrimental BTM changes observed in previous animal/human studies [12,13,22–24,26].

In conclusion, no significant changes were observed in serum markers of bone formation or resorption after six nights of insufficient sleep duration of 5 h/night or subsequent recovery sleep in young healthy men. It is possible that changes in body weight and/or physical activity, which did not occur by design in the current study, may have been mechanistically linked to the changes in BTMs observed in response to insufficient sleep duration in previous studies.

## Figures and Tables

**Table 1:** Participant demographics. Data are presented as mean ± standard deviation (SD) or mean (%).

Age (years)	28.3 ± 5.7
Race/Ethnicity	
Asian/Not Hispanic or Latino	4 (33.3%)
Black or African American/Not Hispanic or Latino	2 (16.7%)
White/Not Hispanic or Latino	6 (50%)
BMI (kg/m^2^)	23.3 ± 2.6
25-hydroxyvitamin D (ng/mL)	29 ± 12.1
Calcium (mg/dL)	9.7 ± 0.3
Creatinine (mg/dL)	0.86 ± 0.10
L-spine BMD (g/cm^2^)	1.073 ± 0.155
L-spine Z-score	−0.2 ± 1.5
Total Hip BMD (g/cm^2^)	1.043 ± 0.137
Total Hip Z-score	0.0 ± 0.9
Apnea-Hypopnea Index (AHI)	7.7 ± 4.2
PSQI Score (at screening)	3.1 ± 0.9
Epworth Sleepiness Scale (at baseline)	4.3 ± 3.0

BMI = body mass index; L-spine = Lumbar spine; BMD = bone mineral density; PSQI = Pittsburgh Sleep Quality Index

**Table 2:** Baseline concentrations and effect of sleep restriction intervention on bone turnover markers (N = 12). Data presented as estimate ± standard error of the estimate (SEE).

	Baseline Biomarker concentration*	Effect of Intervention
	Estimate +/− SEE	Estimate +/− SEE, *p*-value
**PINP (** *μ* **g/L)**	72.1 +/− 4.4	2.7 +/− 4.2, 0.53
**β-CTX (ng/L)**	197 +/− 50	−57 +/− 30, 0.10
**Osteocalcin (ng/mL)**	20.8 +/− 2.0	0.7 +/− 1.6, 0.66

* Baseline biomarker concentration calculated using all data points in first 24-hour sample collection.

**Table 3:** Change in Bone Biomarker Concentrations From Inpatient Through the End of the 3-week Observation Period (N = 11). Data presented as estimate ± standard error of the estimate (SEE)

	Baseline Biomarker concentration**	Effect of Outpatient Observation
	Estimate +/− SEE	Estimate +/− SEE, *p*-value
**PINP (** *μ* **g/L)**	67.8 +/− 6.1	−1.4 +/− 5.8, 0.82
**β-CTX (ng/L)**	571 +/− 90	32 +/− 60, 0.63
**Osteocalcin (ng/mL)**	22.3 +/− 2.4	0.8 +/− 2.1, 0.70

** Baseline biomarker concentration calculated using three morning fasted samples per participant throughout the inpatient stay (one from each 24-hour profile and on day 9 prior to discharge).

**Table 4:** Change in Physical Activity as Measured by Fitbit Charge 3 and ActiWatch2 from (a) Outpatient Week to Inpatient Week, and (b) baseline 24-hour (h) serum collection to sleep restricted 24 h serum collection.

	Steps per Day (Fitbit Charge 3)	Arbitrary Activity Count (ActiWatch2)
(a) Daily average ± standard deviation (SD)		
Inpatient Stay	13,731 ± 3,161	266,043 ± 78,342
Outpatient Run-In Week	14,306 ± 3,052	320,012 ± 80,179
Difference (*p*-value)	−575 ± 648 (*p* = 0.01)	−34,682 ± 32,513 (*p* = 0.005)
Percent Change (*p*-value)	−4.2% ± 4.4% (*p* = 0.01)	−11.4% ± 10.0% (*p* = 0.005)
(b) Average ± SD during 24 h serum profile collection		
Sleep Restricted	12,185 ± 3,360	254,950 ± 65,007
Baseline	12,763 ± 2,711	277,636 ± 82,655
Difference (*p*-value)	−578 ± 3,057 (*p* = 0.53)	−18,343 ± 74,818 (*p* = 0.44)
Percent Change (*p*-value)	−3.3% ± 19.5% (*p* = 0.53)	−3.5% ± 24.5% (*p* = 0.44)

## Abbreviations:

BTM: Bone turnover marker
h: hour
PINP: N-terminal propeptide of type I procollagen
β-CTX: C-telopeptide of type 1 collagen
BMD: bone mineral density
Δ: change
